# Role of Defects on the Particle Size–Capacitance Relationship of Zn–Co Mixed Metal Oxide Supported on Heteroatom‐Doped Graphenes as Supercapacitors

**DOI:** 10.1002/advs.202204316

**Published:** 2022-10-18

**Authors:** Jiajun Hu, Yong Peng, Josep Albero, Hermenegildo García

**Affiliations:** ^1^ Instituto Universitario de Tecnología Química CSIC‐UPV Universitat Politècnica de València‐Consejo Superior de Investigaciones Científicas Universitat Politècnica de València Avda. De los Narajos s/n Valencia 46022 Spain

**Keywords:** defects, doped graphene, energy storage, nanoparticles, supercapacitors

## Abstract

Supercapacitors are considered among the most promising electrical energy storage devices, there being a need to achieve the highest possible energy storage density. Herein small mixed Zn–Co metal oxide nanoparticles are grown on doped graphene (O‐, N‐ and, B‐doped graphenes). The electrochemical properties of the resulting mixed Zn–Co metal oxide nanoparticles (4 nm) grown on B‐doped graphene exhibit an outstanding specific capacitance of 2568 F g^−1^ at 2 A g^−1^, ranking this B‐doped graphene composite among the best performing electrodes. The energy storage capacity is also remarkable even at large current densities (i.e., 640 F g^−1^ at 40 A g^−1^). In contrast, larger nanoparticles are obtained using N‐ and O‐doped graphenes as support, the resulting materials exhibiting lower performance. Besides energy storage, the Zn–Co oxide on B‐doped graphene shows notable electrochemical performance and stability obtaining a maximum energy density of 77.6 W h Kg^−1^ at 850 W Kg^−1^, a power density of 8500 W Kg^−1^ at 28.3 W h Kg^−1^, and a capacitance retention higher than 85% after 5000 cycles. The smaller nanoparticle size and improved electrochemical performance on B‐doped graphene‐based devices are attributed to the higher defect density and nature of the dopant element on graphene.

## Graphical abstract

Supercapacitors hold considerable promise over real energy needs in different applications such as wearable electronic devices and automotive, among others. Here, the structure–performance relationship of Zn and Co mixed metal oxide nanoparticles supported on different doped graphenes, is studied. The origin of the performance differences between the different doping elements on graphene is also investigated in detail.

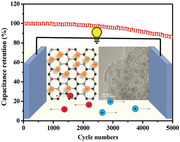

## 1 Introduction

Supercapacitors are considered very promising electrochemical energy storage devices, especially for wearable and portable electronics or, even, electrical and hybrid vehicles,^[^ ^1^ ^]^ as consequence of their capability to provide high power, their long cycle stability, and relative low cost.^[^ ^2^ ^]^

Supercapacitors can be classified according to the energy storage mechanism in i) electric double‐layer capacitors (EDLC), which work on the principle of charge storage by electrostatic charge attraction at the interface between electrolyte and electrode, and ii) pseudocapacitors (PC), which involves charge storage based on reversible surface faradaic reactions. EDLCs are typically composed by carbonaceous materials, exhibiting large surface area, high electrical conductivity, and stability. PCs are typically based on metal oxides or polymers, which can store much more energy via surface redox reactions than EDLC, though they often provide lower power and present low electrical conductivity and stability.^[^ ^3^ ^]^ For those reasons, composites of carbon‐based materials and metal oxides have been pointed out as good candidates to overcome the limitations of their individual components, combining high energy storage with high power and stability.^[^ ^4^ ^]^

Graphene (G), a one‐atom thick sheet constituted by sp^2^ carbons in hexagonal arrangement, has demonstrated unique properties such as high electric and thermal conductivity, high specific surface area (2630 m^2^ g^−1^), and may exhibit a strong interaction with supported metal or metal oxide nanoparticles, as consequence of the overlap of the extended *π* orbitals in G with the d orbitals in metal atoms.^[^ ^5^ ^]^ However, the sp^2^ hybridized orbitals in G lead to delocalization of electrons, and thus, G is considered electrochemically inert. In this regard, heteroatom doped G‐based materials have been more recently used to enhance the capacitor properties of G. Nitrogen has been the most preferred heteroatom for doped G, since it has demonstrated to improve the overall capacitance.^[^ ^6^ ^]^ Alternatively, other heteroatoms, such as B, S, and P, have been also investigated as active components for supercapacitors.^[^ ^7^ ^]^

On the other hand, transition metal oxides are generally considered very convenient candidates as electrode materials in PC as consequence of the variety of oxidation states for redox charge transfer reactions. However, in comparison to single metal oxides, bimetallic oxide materials are more preferred since they can overcome some of the constraints of single metal oxides, such as their low electrical conductivity and electron transfer resistance. Therefore, mixed metal oxides with adequate stoichiometry have been extensively investigated as active components in supercapacitor electrodes, due to the multiple valences of transition metal cations exhibiting low activation energy for electron transfer and enhancing their electrochemical performance for charge storage and release.^[^ ^8^ ^]^ Among the transition metal oxides, Co_3_O_4_ has been exhaustively investigated for supercapacitor applications,^[^ ^9^ ^]^ due to its high theoretical capacitance (3560 F g^−1^) and good electrochemical performance.^[^ ^10^ ^]^ However, in many cases, the observed specific capacitances are far below the theoretical maximum as consequence of the sluggish electrical conductivity, incomplete Faradaic charge of all the Co ions and restricted electrochemical stability during the galvanostatic charge–discharge (GCD) cycles.^[^ ^11^ ^]^ In order to solve these problems, the introduction of a second metal ion, such as Zn^2+^ or Ni^2+^, forming mixed metal oxides, has demonstrated to improve not only their electrical conductivity, but also the capacitance.^[^ ^12^ ^]^ In this regard, ZnCo_2_O_4_ is one of the benchmark materials in supercapacitors,^[^ ^13^ ^]^ exhibiting a high theoretical capacitance (2650 F g^−1^) due to their high electrochemical activity and rich redox reactions.^[^ ^14^ ^]^ However, it still suffers from insufficient electrical conductivity, low surface area, and large capacitance upon decrease cycling.^[^ ^15^ ^]^

In order to circumvent all mentioned issues, mixed metal oxide composites with heteroatom doped G have been proposed as high performing materials for supercapacitor electrodes.^[^ ^16^ ^]^ However, the influence of defects and heteroatom doping on G on the performance of these G‐supported mixed metal oxides as electrodes for supercapacitors still remains mostly unexplored.

In this manuscript, we report the preparation and performance of mixed Zn–Co metal oxide (ZCO) nanoparticles supported on heteroatom doped G. The three doped G used in the present study, O‐doped (OG), N‐doped G (NG), and B‐doped G (BG) were prepared from biomass wastes as precursors. Mixed Zn–Co metal oxide nanoparticles were subsequently deposited on the three defective Gs. It must be highlighted that, unlike the well‐known spinel crystal structure ZnCo_2_O_4,_ the obtained ZCO nanoparticles presented a strong interaction with the doped G support, manifested among other properties on the small average particle size of ≈4 nm on BG and ≈50 nm on NG or OG. In comparison micrometric crystals are typically reported for the standard ZnCo_2_O_4_ material.^[^ ^13^, ^17^ ^]^

The charge storage properties of the new ZCO/G composites have been studied in detail. A remarkable high capacitance (2568 F g^−1^ at 2 A g^−1^) was obtained from ZCO/BG electrodes, while lower capacitance values obtained for ZCO/NG and ZCO/OG electrodes were 1638 and 1484 F g^−1^ at 2 A g^−1^, respectively. These values for ZCO/G composites were higher than those obtained from their individual components. Remarkably, ZCO/BG presented a notable capacitance (640 F g^−1^) at very high current density (40 A g^−1^), which makes this material very convenient for fast‐charge applications in portable devices and vehicles. Moreover, complete supercapacitor devices were fabricated using ZCO/BG as anode and a N‐doped carbon as cathode, obtaining an energy density of 77.6 W h kg^−1^ at 850 W kg^−1^ and a maximum power density of 8500 W Kg^−1^ at 35.1 W h Kg^−1^ energy density. Moreover, ZCO/BG composite has demonstrated to be very stable during 5000 charge–discharge cycles.

The obtained energy storage values are among the highest reported in the literature, including those obtained from ZnCo_2_O_4_/carbonaceous materials composites (see Table S1, Supporting Information). For instance, K. Jiang reported the hydrothermal synthesis of ZnCo_2_O_4_/r‐GO (r‐GO: reduced graphene oxide) composites as supercapacitors. The obtained microscopic ZnCo_2_O_4_ particles were stacked onto r‐GO, and optimized asymmetric devices exhibited a maximum energy of 49.1 W h Kg^−1^ at 400 W Kg^−1^.^[^ ^16^ ^]^ In different example, A. Gaur reported the preparation of g‐C_3_N_4_ supported ZnCo_2_O_4_ electrodes for energy storage applications. Microscopic particles of ZnCo_2_O_4_/g‐C_3_N_4_ composites were obtained. Symmetric supercapacitors were fabricated, obtaining 39 W h Kg^−1^ energy density at 1478 W Kg^−1^ power density.^[^ ^15^ ^]^ These precedents remark the relevance of the herein presented synthetic procedure to obtain small nanoparticles, considered in many applications a key factor to obtain large active surface areas, and, as consequence, improved energy storage properties.

The origin of the improved performance and observed differences between ZCO/BG and ZCO/NG were investigated in detail. The data suggest that the higher density of defects and the nature of the doping element promote in the case of BG large amounts of small (≈4 nm) and narrow size distributed ZCO nanoparticles on its surface, while larger (≈50 nm) and with a wider size distribution nanoparticle were observed on NG. Moreover, impedance spectroscopy measurements revealed that the defective nature also promotes faster charge transfer reactions with the electrolyte in the case of BG. Altogether, enhancing the overall capacitive properties in ZCO/BG over ZCO/NG or ZCO/OG.

## 2 Results and Discussion

### 2.1 Materials Preparation and Characterization

**Scheme 1:**
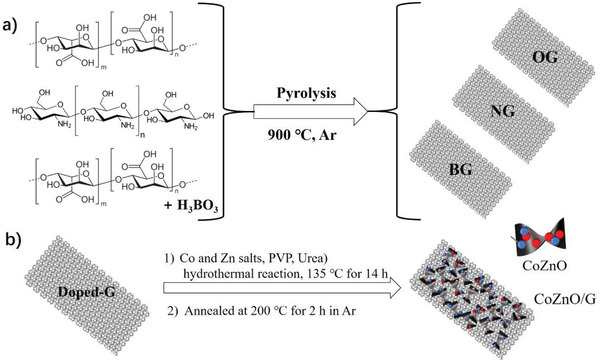
Preparation procedure of a) OG, NG, and BG, and b) the mixed Zn–Co metal oxides—doped G composites, comprising hydrothermal reaction with ZCO precursor and the corresponding doped G (1) and subsequent thermal annealing (2).

The ZCO/G samples are composed by three different graphenes (G) and the mixed Zn–Co metal oxide (ZCO). Sample preparation is schematically illustrated in **Scheme** **1** and described in detail in the Experimental Section. In brief, following reported procedures chitosan and a mixture of alginic acid and boric acid (1:1 wt:wt) were pyrolyzed at 900 °C under Ar atmosphere to obtain NG and BG, respectively.^[^ ^18^ ^]^ Alginate pyrolysis renders, on the other hand, a material similar in composition to OG.^[^ ^19^ ^]^ The chemical composition of the different graphenes was determined by combustion elemental analysis (EA) and the metal content in the ZCO/G composites was determined by inductively coupled plasma optical emission spectroscopy (ICP‐OES). It should be noted that alginic acid and chitosan are polysaccharides having similar structure (see Scheme 1) and the structure and functional groups of the resulting graphenes are similar except for the presence of dopant elements. This similar structure should minimize the influence of the substrate on the particle size of the deposited metal oxide.

Then, ZCO‐G composites were prepared through a two‐step synthesis. In brief, an aqueous solution containing the ZCO precursors (Zn(NO_3_)_2_, Co(NO_3_)_2_) and the different G dispersions (OG, NG, and BG) were submitted to hydrothermal synthesis at 135 °C for 14 h in a Teflon‐lined autoclave. The mixture, also containing urea, should gradually decompose during the hydrothermal synthesis, generating NH_3_ to increase the solution pH value forming the mixed ZCO oxide, while PVP should control the morphology and growth of the nanoparticles becoming deposited on the G. After hydrothermal reaction, the obtained solids were profusely washed with ultrapure MilliQ water and ethanol, and dried at 80 °C overnight. Finally, the composite materials were annealed at 200 °C under Ar atmosphere for 2 h. For comparison purposes, unsupported ZCO oxide was also prepared using identical synthetic procedure, but in the absence of G. Table S2, Supporting Information, summarizes the main analytical data of the samples under study. As can be seen there, the percentage of Co, Zn, and O in ZCO was of 32.1, 36.5, and 24.7 wt%, respectively. These analytical data indicate the formation of mixed Zn–Co oxide with an empiric formula of Zn_1_Co_0.98_O_2.77_, close to ZnCoO_3_ but indicating a considerable degree of O atom vacancies. The Zn and Co ratio can be adjusted by means of the Zn and Co precursors content. Hence, samples with approximately double Zn wt% (Z2CO) or Co wt% (ZC2O) were also prepared (Table S2, Supporting Information). It is worth noticing that ZCO shows very different stoichiometry than the benchmark ZnCo_2_O_4_ spinel structure that is a reference material in supercapacitors. It is also remarkable the large ZCO loadings on the different G that can be achieved in this synthesis, typically near 70 wt%.

**Figure 1:**
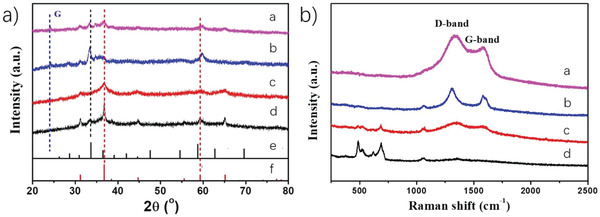
a) XRD patterns of (a) ZCO/BG, (b) ZCO/NG, (c) ZCO/OG, and (d) ZCO. The standard patterns of (e) ZnO (PDF #21–1486) and (f) Co_3_O_4_ (PDF #43‐1003) are also included. b) Raman spectra of (a) ZCO/BG, (b) ZCO/NG, (c) ZCO/OG, and (d) ZCO. Laser excitation 514 nm.

The crystal structure of the prepared samples was analyzed by X‐ray diffraction (XRD). As can be seen in **Figure** **1**, ZCO peaks (31.1°, 33.4°, 36.9°, 44.7°, 59.3°, and 65.3°) can be assigned to the overlap of the standard XRD pattern of Co_3_O_4_ (PDF #43‐1003) (31.2°, 36.6°, 44.8°, 59.2°, and 65.1°) and the main diffraction peak of ZnO (PDF #21‐1486), centered at 33.8°. Interestingly, the observed peaks in ZCO do not exactly match the ones of the standard Co_3_O_4_ and ZnO phases. This shift in the angles of the diffraction peaks has been previously reported in Zn‐doped Co_3_O_4_ and Zn_1 −_ *_x_* Co*_x_* O mixed oxides,^[^ ^20^ ^]^ and it has been attributed to the insertion of Zn^2+^ ions in the Co_3_O_4_ lattice and to the difference in Zn^2+^ and Co^2+^ ionic radii (0.060 and 0.058 nm, respectively). Therefore, XRD provides evidence of the formation of mixed zinc–cobalt oxide. The XRD pattern of the ZCO/G composites present broader and less intense ZCO peaks, together with the broad band characteristic *π*–*π* layer stacking in graphitic carbons near 24° (Figure 1a). The ZCO/OG XRD pattern shows similar reflections than ZCO, with no significant contribution from ZnO at ≈33.8°. In contrast, ZCO/NG XRD pattern shows a main peak at 33.5°, assigned to ZnO species, together with other peaks at 31.2°, 36.1°, and 59.9° due to Co_3_O_4_. In the case of ZCO/BG, its XRD pattern shows similar diffraction pattern than the unsupported ZCO. In conclusion, ZCO crystal structure can be assigned to a mixture of Co_3_O_4_ and ZnO in different proportion depending on the nature of the G support. Interestingly, depending on the different doped G support (OG, NG, and BG), the exposure of some crystal facets could be favored over the others as determined from the differences in the relative intensity of the corresponding diffraction peaks, indicating that the doping in the graphenic support could influence the preferential growth of certain crystal facets of the mixed ZCO metal oxide. The samples containing different Zn and Co ratios (Z2CO/NG and ZC2O/NG) present similar XRD pattern as the related ZCO/NG sample (see Figure S1, Supporting Information), but in the material with larger Zn content (Z2CO) the 33.5° peak can be clearly observed, while in ZC2O this peak was not obvious, indicating that these samples are also constituted by mixed Co_3_O_4_/ZnO oxides, but with different percentages.

The Raman spectra of OG, NG, and BG are shown in Figure S2, Supporting Information. In all cases, the spectra exhibit the typical peaks at 1590 and 1350 cm^−1^, corresponding to the characteristic G and D bands, respectively, in heteroatom‐doped G. The Raman spectrum of ZCO (Figure 1b) shows four peaks located in the low frequency region at 486, 526, 619, and 688 cm^−1^, corresponding to four Raman‐active modes of ZCO.

These Raman shifts are slightly shifted and broader than those of the characteristic Co_3_O_4_ vibrational bands assigned to the *E*_g_, *F*_2g_, *F*_2g_, and *A*_1g_ modes (482, 523, 618, and 691 cm^−1^, respectively).^[^ ^21^ ^]^ It is known that Raman scattering is very sensitive to the material crystalline structure,^[^ ^22^ ^]^ and the observed peaks shift and broadening has been previously reported in Zn‐doped Co_3_O_4_ spinel structure.^[^ ^21^ ^]^ It is worth noticing that the typical Raman shifts of pure ZnO, located at 327, 380, and 437 cm^−1^, could not be observed,^[^ ^23^ ^]^ in agreement with XRD data that show a residual ZnO amount present in ZCO. Additional peak in ZCO at 1057 cm^−1^ could be attributed to residual Co_2_O_3_, which Raman shifts have been reported at 690 and 1080 cm^−1^.^[^ ^24^ ^]^

Raman spectra of ZCO/OG, ZCO/NG, and ZCO/BG are also presented in Figure 1b. These spectra also show the characteristic D and G bands of doped graphenes. However, the four vibrational modes of ZCO can only be observed in ZCO/OG, while these bands are undetectable in ZCO/NG and ZCO/BG. It is worth noticing that the ZCO loading in all these samples is very similar (Table S2, Supporting Information), and therefore, the lack of observation of these vibrational bands could be interpreted as due to the high dispersion and strong interaction of ZCO with the G support with the occurrence of charge density transfer between the semiconductor ZCO and the NG and BG supports.

The surface chemical composition of the composites was further investigated by X‐ray photoelectron spectroscopy (XPS). As can be seen in Figure S3, Supporting Information, the high‐resolution C 1s peaks of OG, NG, and BG are very similar, and the best fitting to individual components show the presence of sp^2^ graphenic C (284.5 eV), sp^3^ C atoms (285.1 eV), and carbon atoms bonded to oxygen with single or double bond (≈286.8 eV) or carboxylic acid (≈289.5 eV) groups. Additionally, XPS N 1s peak in NG was deconvoluted in three individual components, assigned to pyridinic N (398.0 eV), quaternary graphitic N (400.3 eV), and N‐oxides (402.9 eV), respectively. Alternatively, deconvolution of XPS B 1s signal to individual components in BG revealed the presence of BC_3_ (190.5 eV), BC_2_O (191.7 eV), and BCO_2_ (192.1 eV) coordination, respectively. The sp^2^ C—B component (282.8 eV), indicative of the boron substitution in graphene, was also appreciated in the C 1s spectrum of BG, while C atoms bonded to N (285.5 eV) can also be observed in the C 1s peak of NG.

The chemical composition of ZCO was also investigated by XPS. Figure S4a, Supporting Information, shows the characteristic Zn 2p peak doublet corresponding to Zn^2+^. The spin‐orbit coupling of Zn 2p 3/2 and Zn 2p 1/2 peaks appears at binding energy of 1022.3 and 1045.3 eV, respectively. These core level binding energies are shifted from those typical ZnO spectrum.^[^ ^25^ ^]^ However, the separation between them is of 23.0 eV, in good agreement with reference values in literature for ZnO (23.2 eV).^[^ ^25^ ^]^

The XPS Co 2p spectrum shows also two major peaks with binding energies at 781.72 and 797.71 eV, corresponding to Co 2p 3/2 and Co 2p 1/2, respectively. The spin‐orbit splitting of these two peaks is 16.0 eV. Two strong satellite peaks located at 786.91 and 803.44 eV were also observed. The XPS Co 2p peaks were deconvoluted into two different components. The fitting peaks with the binding energies of 781.5 and 797.2 are attributed to Co^3+^, while the peaks at 783.4 and 798.8 eV are ascribed to Co^2+^. These peaks are shifted to higher binding energies compared to those of Co_3_O_4_ (780.0 and 795.1 eV)^[^ ^26^ ^]^ and ZnCo_2_O_4_ (780.8 and 795.9 eV),^[^ ^27^ ^]^ which unlike ZCO exhibits very weak satellites. It has been previously reported that the Co 2p signals shift to higher binding energies when Zn ions are incorporated in Co_3_O_4_ lattice,^[^ ^28^ ^]^ indicating electronic interaction between Zn and Co. Moreover, the intensity of the two satellite peaks also increases with Zn ion content, due to the existence of unpaired electrons in Co^3+^.^[^ ^28^ ^]^

The XPS O1s spectrum can be deconvoluted in four components, 529.5, 530.9, 532.1, and 533.4 eV, corresponding to O species in the mixed metal oxide structure, O—C bonds, oxygen vacancies, and chemisorbed H_2_O, respectively.^[^ ^29^ ^]^

XPS measurements also allowed us to determine the atomic percentage of each element on the surface of ZCO. An atomic Zn:Co ratio near 1:1 was measured, in good agreement with the analytical data obtained from ICP‐OES.

Overall, XRD, Raman, and XPS point out the formation of a Co_3_O_4_ and ZnO phases in ZCO with a large Zn^2+^ content inserted in the Co_3_O_4_ lattice with Co^2+^ and Co^3+^ valence states.

**Figure 2:**
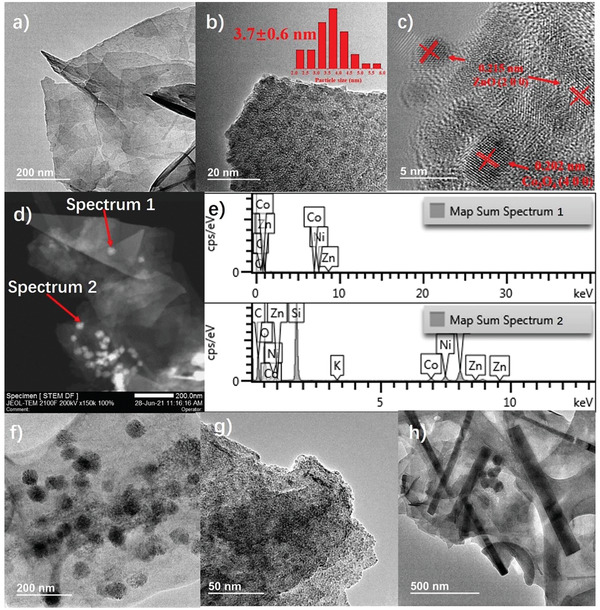
a–c) Selected HRTEM images of ZCO/BG. b) also includes the histogram of ZCO particle size distribution. Red numbers indicate the average particle size and standard deviation after measurement of the diameter of 48 nanoparticles. c) The lattice fringes of the (200) and (400) facets in ZCO have been indicated in red. e) EDS analysis of some nanoparticles in a selected STEM image (d). TEM images of f) ZCO/NG, g) ZCO/BG, and h) ZCO.

The sample morphology and the ZCO size distribution were studied by high‐resolution transmission electron microscopy (HRTEM). The samples exhibit the characteristic 2D morphology of doped graphenes with micrometric lateral size and the typical wrinkles (**Figure** **2**a). The HRTEM images of ZCO/BG show the presence of small nanoparticles homogeneously distributed on the graphene sheets (Figure 2b), with an average particle size of 3.7 ± 0.6 nm, determined by measuring a statistically relevant number of particles. The HRTEM images also allowed us to measure 0.215 and 0.202 nm lattice fringes, very similar to the interplanar distances of the (200) and (400) facets of ZnO and Co_3_O_4_, respectively (Figure 2c). It is worth noticing that no pure ZnO and Co_3_O_4_ crystal phases have been detected in the ZCO XRD patterns. However, the presence of nanometric crystals of these phases cannot be totally discarded. Energy‐dispersive X‐ray spectroscopy (EDS) of representative ZCO/BG scanning transmission electron microscopy (STEM) images (Figure 2d) confirms that individual ZCO nanoparticles supported on BG are constituted simultaneously by Co and Zn. Nanoparticles containing uniquely Zn or Co have not been found. Importantly, HRTEM image of ZCO/NG (Figure 2f) shows notable differences in the ZCO particle size compared with ZCO/BG (Figure 2g). While ZCO small nanoparticles (3.7 nm) are homogeneously distributed over the BG sheets, larger nanoparticles (≈50 nm) can be observed in ZCO/NG. A representative STEM image of ZCO/OG is presented in Figure S5, Supporting Information. As can be seen there, the ZCO particle size in OG is very similar than that of ZCO/NG, about 50 nm or larger. Interestingly, images of ZCO (Figure 2h) shows very large (micrometric size), rod‐like morphology, when this mixed metal oxide is self‐standing and not supported on the doped Gs. The observed differences in ZCO morphology and particle size arising from identical hydrothermal conditions indicate strong interactions between mixed ZCO metal oxide and the different doped G.

In summary, the hybrid ZCO/G samples were composed by doped, defective G sheets containing a large loading of homogeneously distributed nanoparticles. It is worth noticing that despite the large ZCO loading on the G layers, the nanoparticles formed are relatively small, particularly in BG, with a narrow size distribution, highlighting the suitability of doped G as support of these metal oxide nanoparticles and the convenience of this synthetic procedure.

### 2.2 Electrochemical Performance

Electrodes containing the different ZCO‐G composites were fabricated on Ni foam as described in the Experimental Section in order to investigate their electrochemical properties. In the electrochemical measurements, electrodes containing the composite materials were used as working electrodes, while Hg/HgO electrode and Pt wire were used as reference and counter electrodes, respectively. KOH aqueous solution (6 m) was used as electrolyte. For comparison purposes, Co_3_O_4_/NG and ZnO/NG materials were also prepared using identical synthetic procedure (see Experimental Section), and electrodes containing these single metal composites were fabricated and tested.

**Figure 3:**
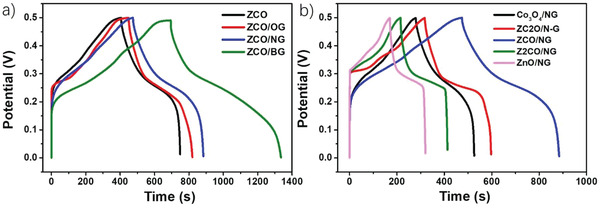
a) GCD curves of ZCO, ZCO/OG, ZCO/NG, and Z2CO/BG. b) GCD curves of ZnO/NG, Co_3_O_4_/NG, ZCO/NG, Z2CO/NG, and ZC2O/NG. Current density 2 A g^−1^.

**Figure** **3**a shows GCD and Figure S6, Supporting Information, the cyclic voltammogram (CV) curves of ZnO/NG, Z2CO/NG, ZCO/NG, ZC2O/NG, and Co_3_O_4_/NG at a scan rate of 50 mV s^−1^ in the potential range from 0 to 0.5 V. As can be seen in Figure S6, Supporting Information, ZCO/NG provides the largest CV area, suggesting higher capacitance than the other samples. The observed peaks in the CV denote pseudo‐capacitive behavior. The higher capacitance of ZCO/NG was confirmed by the GCD curves, obtaining a value of 1638.8 F g^−1^ at 2 A g^−1^. The calculated capacitance values of all studied samples are summarized in Table S3, Supporting Information. As can be seen in Figure 3a, Figure S6, and Table S3, Supporting Information, the capacitance of ZCO/NG is superior to the ones of its individual metal oxides (ZnO and Co_3_O_4_) as well as to Z2CO/NG and ZC2O/NG, with ZCO stoichiometries different to 1:1. It is worth noticing that the anodic peaks in CV from ZnO/NG and Z2CO/NG are very similar, with maximum at 0.404 and 0.406 V, respectively. In comparison, the anodic peaks obtained from Co_3_O_4_/NG and ZC2O/NG are located at 0.362 V, while ZCO/NG is centered at 0.369 V. This is indicating that electrodes containing pure ZnO/NG or Z2CO/NG, with larger Zn content, present higher oxidation potential than that of pure Co_3_O_4_/NG and ZC2O/NG, while the sample with 1:1 stoichiometry (ZCO/NG) is in between.

We have also investigated the effect of the heteroatom doping in the different G in the capacitive properties of these electrodes. For that, CV and GCD curves of ZCO, ZCO/OG, ZCO/NG, and ZCO/BG were acquired at a scan rate of 50 mV s^−1^ in the voltage range from 0 to 0.5 V (Figure 3, Figure S6 and Table S3, Supporting Information, respectively). It can be observed that ZCO, in the absence of heteroatom doped G, exhibited the lowest capacitance (1376 F g^−1^ at 2 A g^−1^). When ZCO was supported on OG a slight capacitance increase was measured (1484 F g^−1^ at 2 A g^−1^). Further increase was achieved with NG (1638.8 F g^−1^ at 2 g^−1^). Remarkably, ZCO/BG presented the highest capacitance of 2568 F g^−1^ at 2 A g^−1^, being almost twice than ZCO and 57% larger than ZCO/NG. Co_3_O_4_/BG and ZnO/BG electrodes were also prepared similarly as ZCO/BG, but in the presence of a single metal, and their electrochemical performance investigated (see Figure S7, Supporting Information). The specific capacitance of these materials, obtained from the GCD curves, are summarized in Table S3, Supporting Information. As can be seen, the specific capacitance of Co_3_O_4_/BG and ZnO/BG materials is higher than that of Co_3_O_4_/NG and ZnO/NG, while ZCO/BG presents the highest capacitance of all samples under study, thus, reconfirming the role of B as dopant element and the superiority of the mixed oxide.

CV and GCD curves of ZCO/BG at different scan rates and current densities can be seen in Figure S8, Supporting Information. As the scanning speed increased, the anodic peak of the CV curves shifted to higher potentials, whereas the cathodic peak shifted toward lower potentials. This is not surprising since the redox reaction rate at the electrode surface can be extremely fast at such a scan rate (50 mV s^−1^), and therefore, different voltages are required for the redox reactions. On the other hand, the GCD curves at different current densities show capacitance decrease with current density increase. This again is typically assigned to the accumulation of charges and ions on the electrode surface. It is worth noticing that even at a high current density of 40 A g^−1^, the specific capacitance obtained with the ZCO/BG electrode is of 640 F g^−1^, which is among the highest capacitance values for these high current densities,^[^ ^30^ ^]^ indicating its superior rate capability. This performance of ZCO/BG is very convenient for supercapacitor fast charge/discharge cycles, especially, for commercial applications in portable devices and automotive.

To further study the ZCO/BG performance a full supercapacitor device was fabricated and studied. This asymmetric supercapacitor consisted of a carbonaceous material derived from chitosan and KOH precursors (CS–KOH) assembled as cathode and ZCO/BG as anode, respectively. The preparation procedure of CS–KOH with a KOH corrosion treatment ensures a large surface area of CS–KOH as previously reported.^[^ ^31^ ^]^ The performance of the CS–KOH electrode was first evaluated by CV and GCD curves in the range from 0 to −1 V at different scan rates and current densities, respectively. The results are presented as Figure S9, Supporting Information.

CV and GCD curves of ZCO/BG//CS–KOH device were measured in the potential window range from 1.5 to 1.8 V at a constant scan rate of 100 mV s^−1^ and 5 A g^−1^, and the curve shape did not change up to a voltage value of 1.7 V, indicating ZCO/BG//CS–KOH asymmetric supercapacitor can operate at this high working voltage (1.7 V) (Figure S10, Supporting Information). The ZCO/BG//CS–KOH asymmetric supercapacitor shows remarkable high energy densities from 77.6 W h Kg^−1^ at 850 W Kg^−1^ power density to 35.1 W h Kg^−1^ at 8500 W Kg^−1^ (see Table S4 and Figure S11a, Supporting Information), indicating excellent energy storage properties. For comparison purposes, asymmetric devices containing ZCO/NG and CS–KOH as anode and cathode, respectively, were also fabricated. The GCD curves at different current densities are presented in Figure S10b, Supporting Information and the obtained values of energy density and power density summarized in Table S4, Supporting Information. As can be seen there, the energy storage characteristics of ZCO/NG//CS–KOH are lower than that of ZCO/BG//CS–KOH, in good agreement with their respective capacitive characteristics.

**Figure 4:**
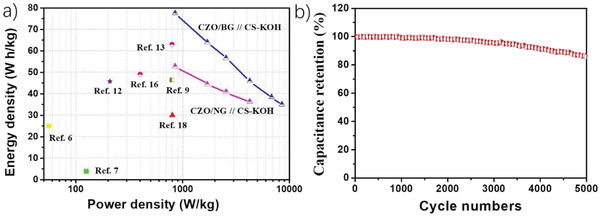
a) Ragone Plot with specific energy and power for different related materials in the literature. b) Capacitance retention over 5000 charge–discharge cycles using ZCO/BG//CS–KOH device at the voltage window of 1.7 V and current density of 5 A g^−1^.

The energy density and power density values of ZCO/BG//CS–KOH were compared with related materials in a Ragone plot (**Figure** **4**a). Table S1, Supporting Information, provides comparison of the performance parameters of these reported devices. In the best of our knowledge, the asymmetric supercapacitor based on ZCO‐BG is among the most efficient devices reported in the literature, confirming its excellent energy storage properties.

Besides the efficient energy storage properties, ZCO/BG//CS–KOH has demonstrated outstanding cycling stability. A 5000‐cycle performance of the asymmetric supercapacitor at a current density of 5 A g^−1^ is presented in Figure 4b. The performance remained nearly constant during the first 1000 cycles, while only a minor performance degradation taking place along the other 4000 cycles, retaining 85.7% of the performance after 5000 cycles.

Taking advantage of the remarkable performance of ZCO/BG//CS–KOH, we have built a quasi‐solid‐state, flat, flexible supercapacitor of 1 × 3 cm^2^, using poly vinyl alcohol (PVA)–KOH gel electrolyte (see Experimental Section for further details and Figure S12, Supporting Information). The CV and GCD curves obtained from ZCO/BG//PVA–KOH//CS–KOH are presented in Figure S13, Supporting Information, and this exhibits a capacitance of 158.7 F g^−1^ at 2 A g^−1^, and an energy density of 49.6 W h Kg^−1^ at power density of 1500 W Kg^−1^. Moreover, measurements of the GCD at 4 A g^−1^ upon different mechanical stress (Figure S14, Supporting Information) revealed very similar behavior, highlighting the robustness of this device.

### 2.3 Origin of the Pseudocapacitive Properties of ZCO/BG

We were interested in gaining understanding on the origin of the improved energy storage properties of ZCO/BG over ZCO/NG. Pumera and co‐workers have previously reported that B‐doped G exhibits higher electrical conductivity than N‐doped G.^[^ ^32^ ^]^ However, the role of B or N doping in the G capacitance has been lately questioned by the same authors,^[^ ^33^ ^]^ and the distinctive structural differences in terms of density of defects between N‐ and B‐doped G has been proposed as the determining factor for their different capacitive properties, rather than the type or heteroatom content.

**Figure 5:**
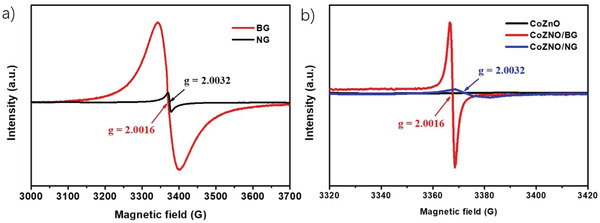
EPR spectra of a) NG and BG and b) ZCO/NG and ZCO/BG at room temperature.

The ratio of the intensity of the D and G Raman peaks (*I*_D_/*I*_G_) is often used as quantitative analysis of the G defectivity. In the present work, the Raman spectra presented in Figure S2, Supporting Information, allowed us to determine an *I*_D_/*I*_G_ ratio for NG and BG of 0.75 and 0.81, respectively, indicating larger concentration of defects in BG. Moreover, the defect nature in NG and BG has been investigated by electron paramagnetic resonance (EPR). As can be seen in **Figure** **5**, the larger EPR signal in BG indicates a higher concentration of radicals that are associated to defects. The observed EPR signal in BG at *g* ≈ 2.0016 can be assigned based on the literature to localized *π* electrons trapped at defects or atom vacancies, indicative of extended sp^2^ domains,^[^ ^34^ ^]^ while the *g* ≈ 2.0032 in NG has been attributed to the formation of C‐related dangling bonds.^[^ ^35^ ^]^ Thus, BG contains a larger density of defects consisting in trapped electrons, favoring higher conductivity, while NG present lower density of defects that in addition should be less mobile because they are associated to sp^2^ carbon related radicals. The different EPR signals in BG and NG supports were consistent with the ones observed in ZCO/NG and ZCO/BG composites, indicating that after the mixed metal oxide deposition, the characteristic defects in NG and BG remained mostly unchanged.

In order to further investigate the observed differences in capacitance between ZCO/NG and ZCO/BG, the high‐resolution XPS Co 2p peaks of these samples were analyzed in more detail. For comparison purposes, the XPS Co 2p of ZCO was also included in the comparison, together with the ZCO/NG and ZCO/BG spectra in Figure S15, Supporting Information. As can be seen there, the Co 2p 3/2 peaks in ZCO/NG and ZCO/BG can be deconvoluted in two different components, assigned to Co^3+^ and Co^2+^ species, together with the corresponding prominent satellite peaks, in good agreement with the measured spectrum of ZCO. However, the Co 2p peaks are negatively shifted 1.23 and 1.71 eV in ZCO/NG (780.49 eV) and ZCO/BG (780.01 eV), respectively, from the binding energy measured for ZCO (781.72 eV). It is worth noticing that these binding energies for the Co 2p peaks in ZCO/NG and ZCO/BG are very different from the reported values of Co metal (778.2 eV) and Co—C bonds at 778.47 eV.^[^ ^36^ ^]^ Therefore, the formation of Co metal and Co_2_C species that could also be observed by XRD or TEM can be ruled out. The observed chemical shifts of Co 2p have been attributed to an enhancement in electron density of the oxidized Co species when they are in contact with the doped G. Similar detailed study of high‐resolution XPS has also shown a shift in the Zn 2p peaks in ZCO/NG (1021.15 and 1044.28 eV) and ZCO/BG (1021.02 and 1044.11 eV), which are also 1.11 and 1.24 eV down shifted from the measured values for Zn 2p in the reference ZCO sample (1022.26 and 1045.36 eV), indicating similar electron density transfer from the doped G to the oxidized Zn. Notice that the O content in ZCO/NG and ZCO/BG materials is lower than that of ZCO (Table S2, Supporting Information), despite NG and BG also have O atoms, suggesting a partial reduction of the ZCO material with the generation of O vacancies (detected in XPS O 1s of the samples) in the hydrothermal synthesis of ZCO/doped G composites, in agreement with the obtained XPS data. Furthermore, regarding the influence of the dopant element on G, the Co 2p 3/2 peak in ZCO/BG presents a 0.48 eV more negative shift than that in ZCO/NG, suggesting a higher electron density transfer from BG to ZCO than from NG. This can be correlated with the higher density of defects assigned to *π* electrons in BG defects, as observed in the EPR measurements.

The differences in terms of density of defects and electron transfer density observed by EPR and XPS can derive for the different ZCO crystal growth, as suggested by the XRD patterns and HRTEM images presented in Figures 1 and 2. In this regard, larger ZCO nanoparticles (≈50 nm) have been observed in ZNO/NG, while ZCO small nanoparticles (≈4 nm), with narrow size distribution, have grown in BG sheets. It is worth noticing that the nanoparticles size plays a decisive role in different fields such as heterogeneous catalysis, electrocatalysis, and energy storage. It is well accepted that small nanoparticles are characterized by their high surface to volume ratio and large number of exposed surface atoms. Therefore, smaller nanoparticles have typically demonstrated enhanced activity in different applications. In the present study, the observed differences in ZCO particle size arising from identical hydrothermal conditions indicates the different interactions, due to the different G defectivity, between the mixed ZCO metal oxide and the different doped G.

A direct consequence from the observed differences in particle size could be a different electrochemically active surface area (ECSA) that rather than the specific surface area determined by isothermal gas adsorption is the relevant parameter to understand the relative performance of this series of samples. CV of ZCO/NG and ZCO/BG at different scan rates (5–20 mV s^−1^) allowed us to determine the ECSA by measuring double‐layer capacitance (*C*_DL_). As shown in Figure S16, Supporting Information, ZCO/NG and ZCO/BG presented *C*_DL_ of 1.07 and 1.57 mF g^−1^, respectively. Hence, one of the reasons of the enhanced capacitance in ZCO/BG can be attributed to its higher ECSA as consequence of the smaller ZCO size. Since the synthesis conditions are identical, the differences in ECSA are probably derived from the higher density of defects in preformed BG and its different nature, promoting stronger interaction with the nascent mixed metal oxide than in the case of NG. This specific interaction with nascent ZCO should favor the formation of a larger number of seeds, resulting in the growth of a larger number of small ZCO nanoparticles compared to in the NG surface.

On the other hand, the observed differences depending on the doped G are also reflected in electric behavior. Electrochemical impedance spectroscopy (EIS) measurements in ZCO, ZCO/NG, and ZCO/BG were performed and the results are presented as Figure S17, Supporting Information. The obtained Nyquist plots consist of a semicircle and a line. Typically, semicircle radius is related to the electrical resistance at the electrode–electrolyte interface (*R*_CT_), while the line at lower frequencies can be attributed to the Warburg factor (*W*), which is related with ions diffusion coefficient. These parameters can be obtained from the best fitting of the experimental data to a model circuit. The model circuit together with the calculated parameters from the Nyquist plots of these samples presented in Table S6, Supporting Information. As can be seen in that Table, the *R*_CT_ in ZCO/BG (2.10 Ω cm^−2^) is lower than that of ZCO/NG (4.10 Ω cm^−2^) and ZCO (3.44 Ω cm^−2^), indicating improved electronic conductivity. Moreover, the lower *W* values suggest a larger ion diffusion coefficient in ZCO/BG than in ZCO/NG and ZCO. These data would indicate that BG is not only contributing in the growth of ZCO small nanoparticles, but also improving the charge transfer and ion diffusion with the electrolyte, exhibiting, in overall, enhanced capacitive properties than ZCO/NG.

**Figure 6:**
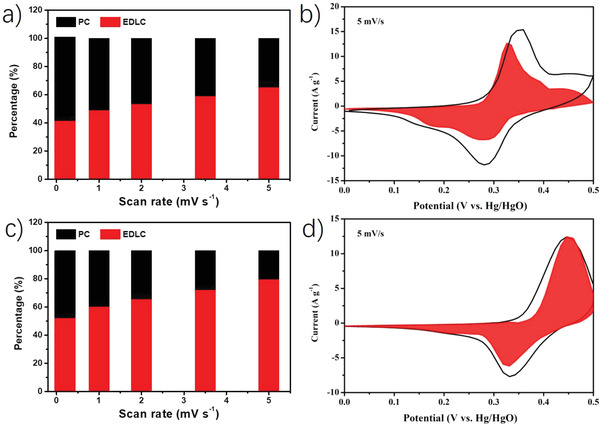
Percentages of EDLC and PC contributions at different scan rates for a) ZCO/BG and c) ZCO/NG. EDLC‐ and PC‐controlled contributions in b) ZCO/BG and d) ZCO/NG electrodes obtained from CV profile at 5 mV s^−1^.

In order to further investigate the differences in charge storage mechanism between ZCO/BG and ZCO/NG, we have calculated the PC versus EDLC contributions. For that, we have measured CV curves of these samples at scan rates from 0.2 to 5 mV s^−1^ (Figure S18, Supporting Information), and analyzed the obtained peak currents (*i*) and scan rates (*v*) of these CV curves using Equation (1).

(1) $$i=av^{b}$$

where *a* and *b* are constant parameters. It has been previously reported that *b* value of 0.5 corresponds to EDLC‐controlled charge storage mechanism, while *b* value of 1 is attributed to PC‐controlled mechanism. The calculated *b* values in anionic and cathodic peaks (Figure S18, Supporting Information) are 0.77 and 0.64; and 0.72 and 0.67, for ZCO/BG and ZCO/NG, respectively. These intermediate values indicate that in both materials the EDLC and PC mechanisms are contributing to the charge storage. More detailed and accurate PC‐controlled contributions can be quantitatively determined by Equation (2).

(2) $$i\left(v\right)=k_{1}v+k_{2}v^{1/2}$$

where *k*_1_ *v* refers to PC, while *k*_2_ *v* ^1/2^ corresponds to EDLC mechanism. As can be seen in **Figure** **6**, the EDLC contribution (red region) is of 41% and 48% of the total current at 0.2 mV s^−1^ scan rate for ZCO/BG and ZCO/NG, respectively, while 65% and 79% are achieved at 5 mV s^−1^. These results are in good agreement with the highest ECSA in ZCO/BG, as consequence of the smallest nanoparticle size, providing higher electrochemical active surface for Faradaic reactions, and therefore, higher a PC contribution. On the contrary, the lower ECSA in ZCO/NG, due to the higher ZCO particle size, give rise to a lower PC contribution in the overall energy storage mechanism.

It is worth noticing that ZCO/NG sample present redox peaks at higher potential than that of ZCO/BG. In order to rationalize this result, it must be commented that both samples contain mixed Zn and Co oxide nanoparticles with Co and Zn molar ratios close to 1. However, the XRD patterns of ZCO/NG and ZCO/BG, presented in Figure 1, show a sharper and more intense peak related to the ZnO phase in ZCO/NG than in ZCO/BG, suggesting a higher crystallinity of ZnO in ZCO/NG. On the other hand, it has been previously reported that the redox potential in ZnO is higher than that of Co_3_O_4_.^[^ ^37^ ^]^ Therefore, we conclude that the larger redox potential in ZCO/NG with respect to ZCO/BG could be the consequence of the higher contribution of larger ZnO crystals in the ZCO/NG sample.

## 3 Conclusion

Precedents in the literature have reported that the combination of mixed metal oxides and doped Gs renders composites with a high performance as electrodes for supercapacitors. Herein we have shown that wise selection of the dopant element can increase further this perform. Thus, Zn–Co mixed metal oxide nanoparticles have been deposited by hydrothermal synthesis on three heteroatom doped G observing a large influence of the dopant element on the supercapacitor performance of ZCO nanoparticles depending on the doped G support. ZCO/BG presents the highest specific capacitance of 2568 F g^−1^ at 2 A g^−1^. Moreover, supercapacitors containing ZCO/BG and CS–KOH as anode and cathode, respectively, have demonstrated 86% capacitance retention after 5000 cycles and a maximum energy density of 77.6 W h kg^−1^ at 850 W kg^−1^. The origin of the improved performance of ZCO/BG has been attributed to the nature of the dopant element, and to larger density of defects in BG, resulting in the formation of small ZCO nanoparticles homogeneously distributed on BG surface with a strong interaction with the BG support. Under identical synthetic conditions larger nanoparticles are formed in NG. Moreover, lower charge transfer resistance has been measured in ZCO/BG than in ZCO/NG, indicating that the BG support is also contributing to enhance the electronic and ionic conductivity of this composite. The electrochemical performance of ZCO/BG for charge storage, combining double layer and pseudo‐capacitance mechanisms, ranks this composite at the top of the list of efficient components for the preparation of supercapacitors with high specific power and capacitance.

## 4 Experimental Section

### Chemicals

Commercially available reagents were purchased from Sigma‐Aldrich and used without further purification.

### NG Synthesis

N‐doped graphene (NG) was obtained as reported by pyrolysis of chitosan powder in horizontal electrical furnace under Ar flow (200 mL min^−1^) at 900 °C for 2 h. The heating rate was 2 °C min^−1^.

### BG Synthesis

B‐doped graphene was obtained as reported by pyrolysis of equal amounts of alginic acid and H_3_BO_3_ under Ar flow (200 mL min^−1^) at 900 °C for 2 h. The heating rate was 2 °C min^−1^.

### OG Synthesis

OG was obtained as reported by pyrolysis of alginic acid under Ar flow (200 mL min^−1^) at 900 °C for 2 h. The heating rate was 2 °C min^−1^.

### ZCO/G, Z/G, and C/G Syntheses

First, 20 mL of the different G dispersions (1 mg mL^−1^) was prepared by sonicating at 700 W for 2.5 h. Then, 0.6 mmol of Co(NO_3_)_2_·6H_2_O, 0.6 mmol Zn(NO_3_)_2_·6H_2_O, 16 mg PVP, and 1.2 mmol of urea were dissolved in 22 mL deionized water. After that, 3 mL of G dispersion were mixed into the above solution and introduced into the Teflon‐lined autoclave (for the preparation of the ZCO sample without G, 3 mL of deionized water was added instead). The hydrothermal reaction was carried out at 135 °C for 14 h. After that, the solids were profusely washed with water and ethanol by filtration, and dried at 60 °C overnight. Finally, the obtained materials were annealed under Ar atmosphere at 200 °C for 2 h. Z/G and C/G were prepared following exactly the same procedure, but using only the amount of either zinc or cobalt nitrate in the preparation of the material. The ZCO/G, Z/G and C/G mass loading is ≈1.1–1.3 mg cm^−2^ in all electrodes.

### Chitosan–KOH Synthesis

2 g chitosan (CS) was pyrolyzed under Ar atmosphere at a heating rate of 2 °C min^−1^ up to 700 °C for 1.5 h. Then, 650 mg of the obtained powder and 1.3 g KOH were mixed in 100 mL ethanol. Then, the solvent was evaporated at 75 °C. The obtained powder was pyrolyzed using identical conditions. The final solids were washed several times with water by filtration to remove the KOH excess and dried at 60 °C overnight.

### Sample Characterization

XRD patterns were obtained in a Philips XPert diffractometer (40 kV and 45 mA) equipped with a graphite monochromator employing Ni‐filtered Cu K*α* radiation (1.541178 Å). The chemical analysis was determined by ICP‐OES (iCAP 7400, Thermo Scientific, Waltham, MA, USA). HRTEM images were recorded in a JEOL JEM 2100F under an accelerating voltage of 200 kV. Samples were prepared by applying one drop of the suspended material in ethanol onto a carbon‐coated copper TEM grid and allowing it to dry at room temperature. Raman spectra were collected with a Horiba Jobin Yvon‐Labram HR UV–visible–NIR (200–1600 nm) Raman Microscope Spectrometer using a 514 nm laser. The chemical composition of the samples was determined by combustion chemical analysis by using a CHNS FISONS elemental analyzer. X‐ray photoelectron spectra (XPS) were measured on a SPECS spectrometer equipped with a Phoibos 150 MCD‐9 detector using a non‐monochromatic X‐ray source (Al) operating at 200 W. Before spectrum collection, samples were evacuated in the prechamber of the spectrometer at 1 × 10^−9^ mbar. The measured intensity ratios of the components were estimated from the area of the corresponding peaks after nonlinear Shirley‐type background subtraction and corrected by the response factor of the spectrometer for each element. EPR spectra were recorded in a Bruker EMX instrument operating at frequency 9.80 GHz, sweep width 30.6 G, time constant 80 ms, and microwave power 200 mW.

### Electrochemical Measurements

ZCO/G electrodes were prepared by performing the ZCO/G synthesis into the Teflon‐lined autoclave in which a 1 × 3 cm Ni foam was previously introduced.

CS–KOH electrodes were fabricated mixing CS–KOH powder, carbon black, and PVDF at 8:1:1 ratio, and grinding the mixture in an Agatha mortar using *N*‐methylpyrrolidone (NMP) and ethanol as solvents. The obtained slurry was then brushed onto a 1 cm^2^ piece of nickel foam and dried overnight at 60 °C.

In the electrochemical measurements, the as‐fabricated electrode, Hg/HgO electrode, and a platinum wire were used as the working, reference and counter electrode, respectively. The 6 m KOH was used as electrolyte. For complete supercapacitor device measurements, the ZCO/G/NF, CS–KOH/NF, and glass microfiber filters were used as positive electrode, negative electrode, and separator, respectively.

Cyclic voltammograms (CVs), GCD, and electrochemical impedance spectra (EISs) were measured on a Gamry potentiostat (Interface 5000E) in three‐electrode configuration. CVs were recorded in potential scan rate range of 10–50 mV s^−1^. EISs were recorded with a frequency range of 0.01 Hz–100 kHz. The capacitance of electrodes (*Cs*) was calculated based on the discharge time in GCD according to formula:

(3) $$Cs=\frac{I\times \Delta t}{m\times \Delta V}$$

where *I* (A) represent discharge current, *ΔV* (V) is the potential window, *Δt* represents discharge time(s), *m* (g) is the accurate weight of active material on working electrode.

For the asymmetric supercapacitor, the negative electrode was obtained by coating a mixture of CS–KOH and PVDF with a percentage ratio of 90 versus 10 wt% onto a nickel foam. The mass loading of active materials used for anode and cathode must abide the charge balance relationship (Q+ = Q−) as following equation:

(4) $$\frac{M_{+}}{M_{-}}=\frac{C_{-}\times \Delta V_{-}}{C_{+}\times \Delta V_{+}}$$

Therefore, the necessary amount of CS–KOH in each cathode, in order to store the same number of charges than the anodes for each supercapacitor, was calculated.

The energy density (*E*) and the power density (*P*) of the as‐obtained supercapacitor were evaluated by varying current densities. The values can be achieved by the following formulae:

(5) $$C_{\mathrm{ASC}}=\frac{I\times \Delta t}{M\times \Delta V}$$

(6) $$E=\frac{C_{\mathrm{ASC}}\times \Delta V^{2}}{2}$$

(7) $$P=\frac{E}{\Delta t}$$

the symbols *I*, *Δt*, *ΔV*, and *M* separately represent constant discharge current (A), discharge time (s), potential window (V), and mass (g) of the all active material.

### Quasi‐Solid‐State, Flexible Device Assembly

For the gel electrolyte preparation, 6 g of PVA powder were dissolved in 50 mL of MilliQ water at 85 °C. Alternatively, 6 g of KOH was also dissolved in 10 mL of MilliQ water and added to the PVA solution. The obtained PVA–KOH gel electrolyte was spread on a plastic wrap and allowed dried at room temperature. ZCO/BG and CS–KOH inks were deposited on Ni foam as previously described. ZCO/BG cathode, PVA–KOH electrolyte and CS–KOH anode were sandwiched and sealed using Metlonix 1170‐60 thermoplastic.

### Statistical Analysis

The ZCO average particle size together with the standard deviation was obtained after measuring a statistically relevant number of nanoparticles (*n* = 48) using the following equations:

(8) $$A=\frac{\sum D}{n};\;SD=\sqrt{\frac{\sum {\left(D-A\right)}^{2}}{n}}$$

where, *A* is the average particle size, *D* is the measured nanoparticle diameter in nm, *n* is the number of accounted nanoparticles and *SD* is the standard deviation. The data is presented as mean ± *SD*.

## Conflict of Interest

The authors declare no conflict of interest.

## Supporting information

Supporting Information

## Data Availability

The data that support the findings of this study are available from the corresponding author upon reasonable request.
