# Supplementary material for: Role of Defects on the Particle Size–Capacitance Relationship of Zn–Co Mixed Metal Oxide Supported on Heteroatom‐Doped Graphenes as Supercapacitors
Source: Adv Sci (Weinh). 2022 Oct 18;9(34):2204316. doi: 10.1002/advs.202204316 (PMC9731690; doi:10.1002/advs.202204316)
Supplement: Supplementary file 1 — Supporting Information [file ADVS-9-2204316-s001.pdf]

Supporting Information

Role of defects on the particle size-capacitance relationship of Zn-Co mixed metal oxide supported on heteroatom doped graphenes as supercapacitors

Jiajun Hu, Yong Peng, Josep Albero, and Hermenegildo García**

Table S1. Summary of some reported values of energy density and power density, as well as the ones obtained in this work. These values are also included in Ragone plot in Figure 4 in the main text.

Reference	Electrode	Energy density (Wh/g)	Power density (W/Kg)
[1]	Co_3O_4/N-RGO	25	56
[2]	$ZnCo_2O_4$-rGO	49.1	400
[3]	$ZnCo_2O_4$	63	795.5
[4]	MOF-Derived Co_3O_4	46.5	790.7
[5]	boron-doped graphene	3.86	125
[6]	Co-Zn Mixed Oxide/Hydroxide	45.8	208
[7]	CoZn layered double hydroxide (LDH) supported on carbon fiber paper (CFP)	30	800
This work	ZCO/BG	77.6	850
This work	ZCO/NG	52.8	850

Table S2. Summary of the chemical composition of the different samples

	Co(wt.%)	Zn(wt.%)	B(wt.%)	N(wt.%)	O(wt.%)	C(wt.%)
OG	-	-	-	-	7.6	92.2
NG	-	-	-	6.5	15.0	77.8
BG	-	-	8.8	-	29.0	60.2
ZCO	32.1	36.5	-	-	24.7	5.4
ZCO/NG	28.0	27.4	-	0.3	16.7	26.8
ZC2O/NG	34.2	16.9	-	0.4	22.2	25.4
Z2CO/NG	16.7	32.0	-	0.3	21.0	29.2
ZCO/OG	25.6	26.0	-	-	20.1	27.6
ZCO/BG	28.6	23.3	0.07	-	15.4	30.9

Table S3. Summary of the capacitance of different samples.

**Sample**	**Specific capacitance (F/g)**	**Current density (A/g)**
Co_3O_4/NG	984	2
Co_3O_4/BG	1192	2
Z2CO/NG	1118.8	2
ZCO/NG	1638.8	2
ZC2O/NG	791.2	2
ZnO/NG	598	2
ZnO/BG	704	2
ZCO	1376	2
ZCO/OG	1484	2
ZCO/BG	2568	2
ZCO/BG	2230	3
ZCO/BG	1504	8
ZCO/BG	1320	10
ZCO/BG	880	20
ZCO/BG	720	30
ZCO/BG	640	40

Table S4. Summary of the energy density and power density of ZCO/BG // CS-KOH device at 1.7 V

**Energy density (Wh/kg)**	**Power density (W/kg)**
77.6	850
64.1	1700

56.7	2550
46.0	4250
38.5	6800
35.1	8500

Table S5. Summary of the energy density and power density of ZCO/NG // CS-KOH device at 1.7 V

**Energy density (Wh/g)**	**Power density (W/Kg)**
52.8	850
44.5	1700
40.9	2550
36.4	4250
31.7	6800
28.3	8500

Table S6. Summary of the parameters obtained from the Nyquist plot fitting of the samples to an equivalent circuit.

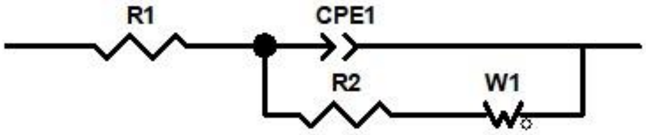

Modeled Values	ZCO/BG	ZCO/NG	ZCO
R1 ($\Omega\cdot cm^2$)	1.03	0.48	0.49
CPE1-T (F/cm^2)	$7.45\cdot10^{-3}$	$5.09\cdot10^{-3}$	$7.79\cdot10^{-3}$
CPE1-P (F/cm^2)	0.82	0.81	0.76
R2 ($\Omega\cdot cm^2$)	2.10	4.10	3.44
W1-R ($\Omega\cdot cm^2$)	0.13	2.04	7.26
W1-T ($\Omega\cdot cm^2$)	0.04	1.44	4.44
W1-P ($\Omega\cdot cm^2$)	0.42	0.43	0.35

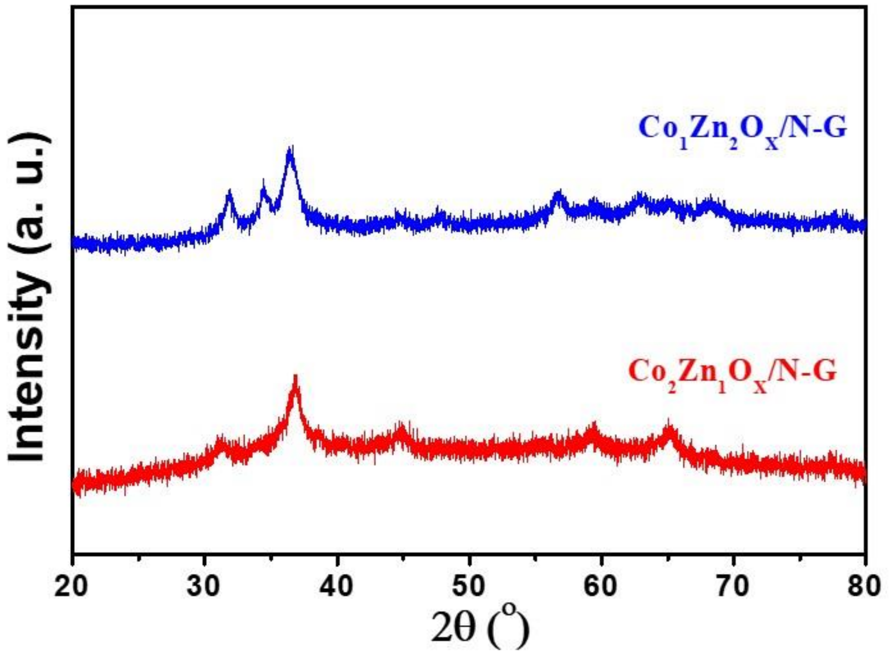

Figure S1. XRD patterns of Z2CO/NG and ZC2O/NG.

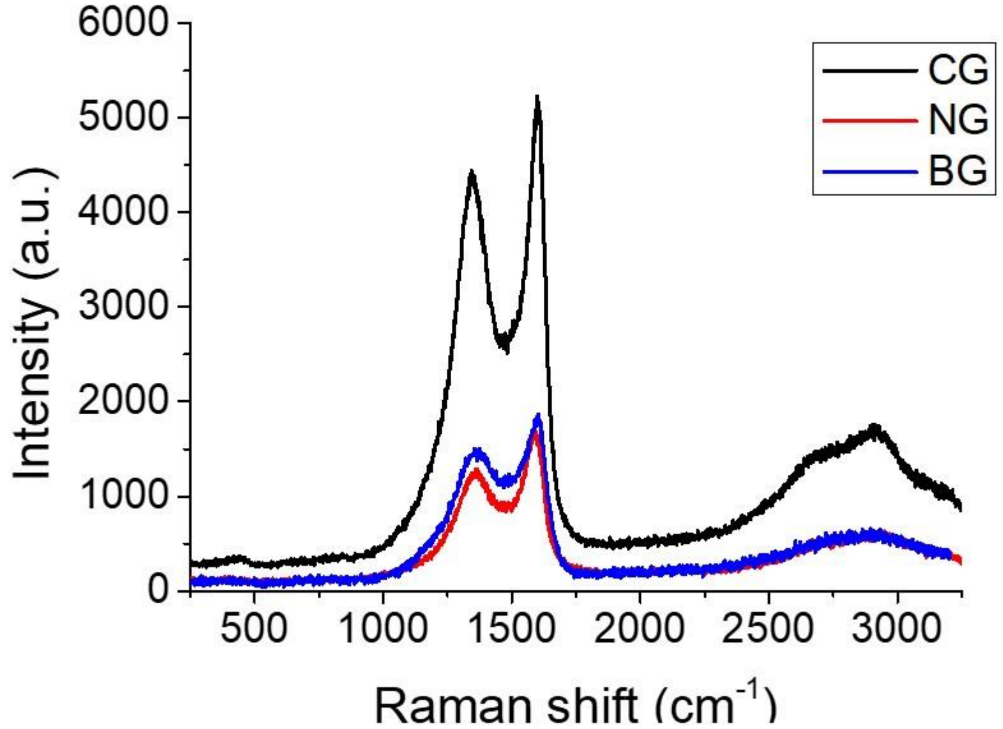

Figure S2. Raman spectra of CG, BG and NG. Laser excitation 514 nm.

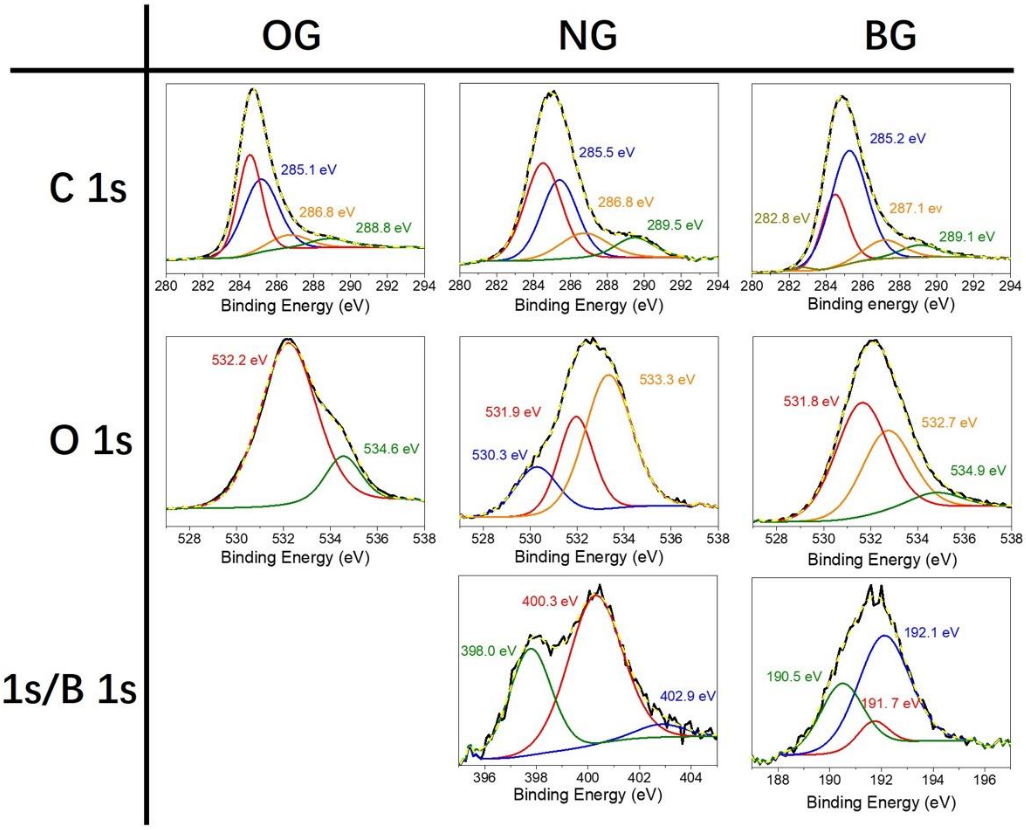

Figure S3. XPS core-level spectra of C 1s, O1s, N1s and B 1s of OG, NG and BG.

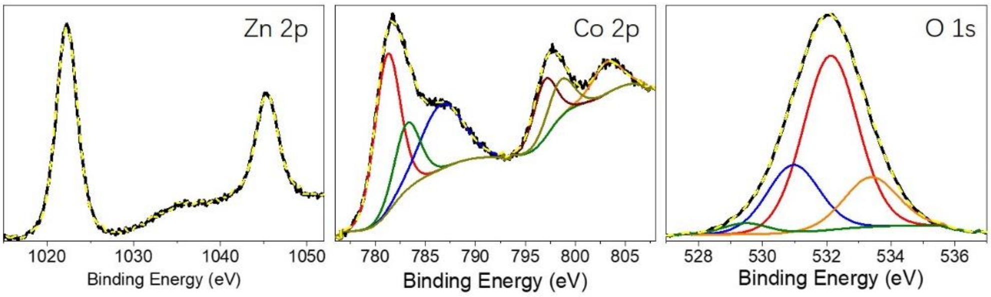

Figure S4. XPS core-level spectra of Zn 2p, Co 2p and O 1s of ZCO.

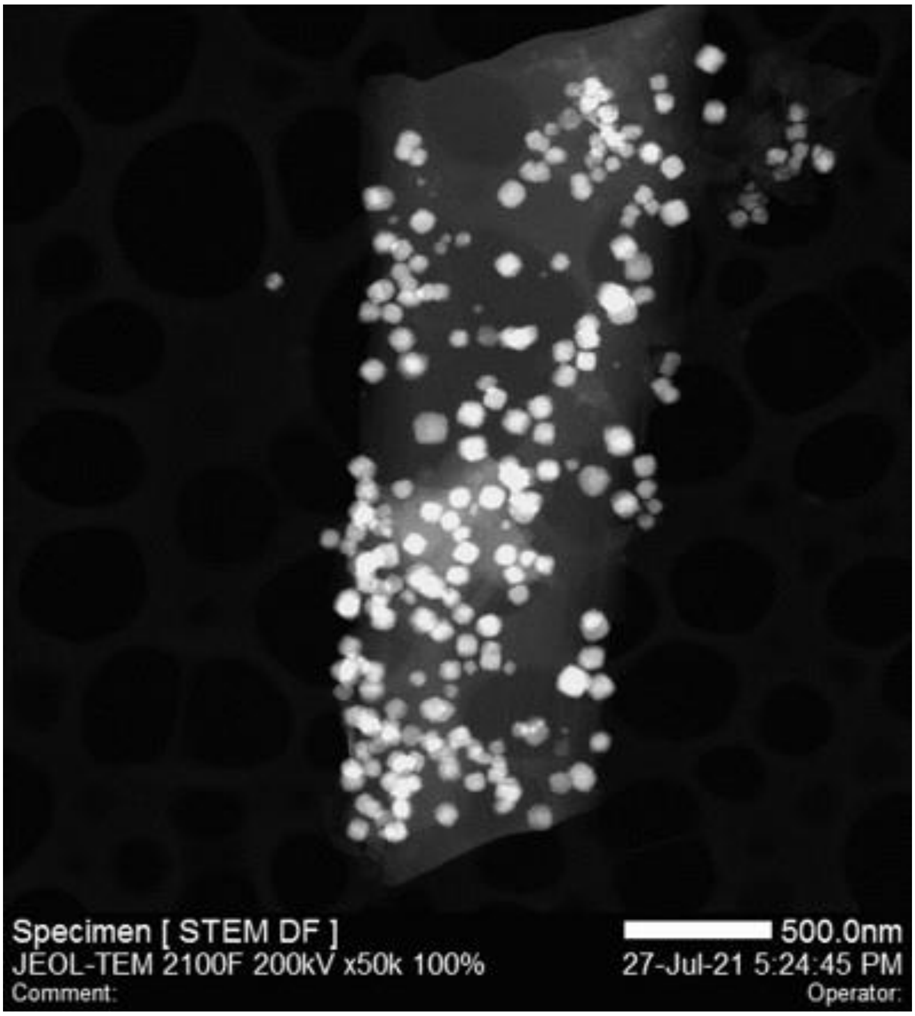

Figure S5. STEM image of ZCO/OG.

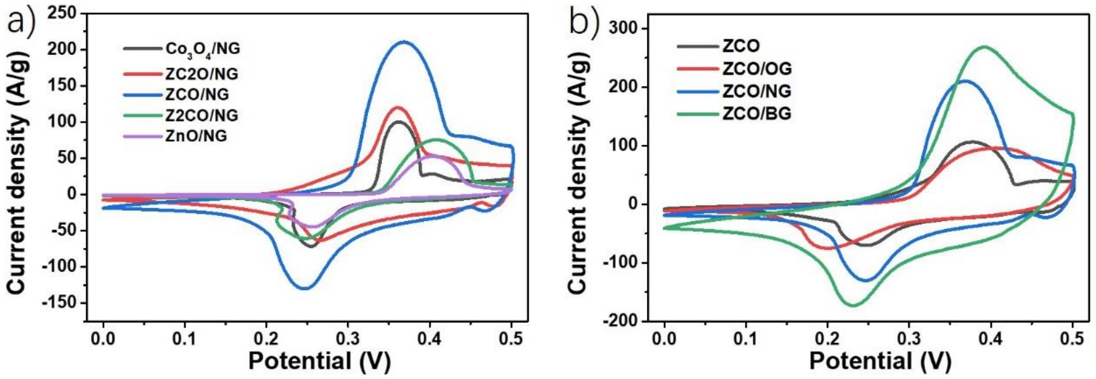

Figure S6. (a) CV curves of ZnO/NG, Co_3O_4/NG, ZCO/NG, Z2CO/NG, and ZC2O/NG. (b) CV curves of ZCO, ZCO/OG, ZCO/NG and ZCO/BG. Scan rate 50 mV/s.

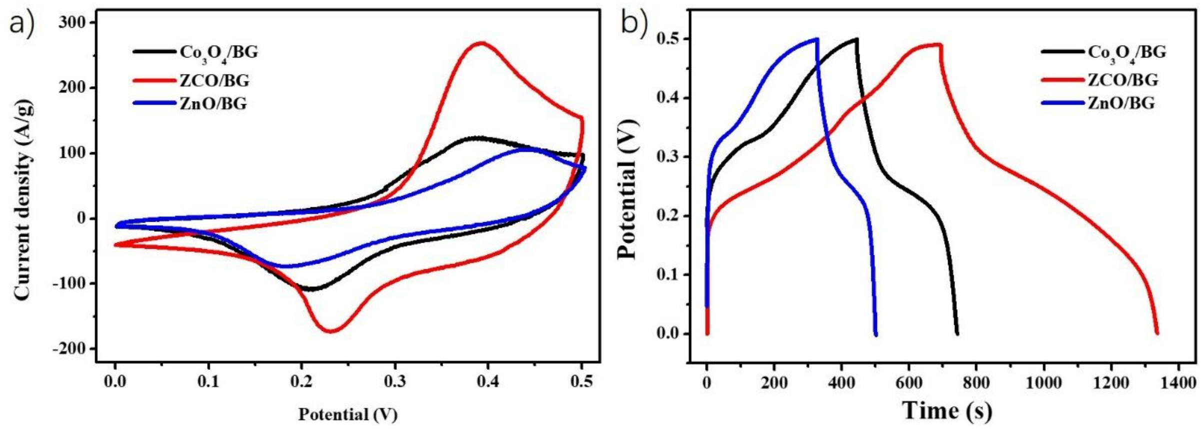

Figure S7. (a) CV and (b) GCD curves of Co_3O_4/BG, ZCO/BG and ZnO/BG measured at 50 mV/s scan rates and 2 A/g current density, respectively.

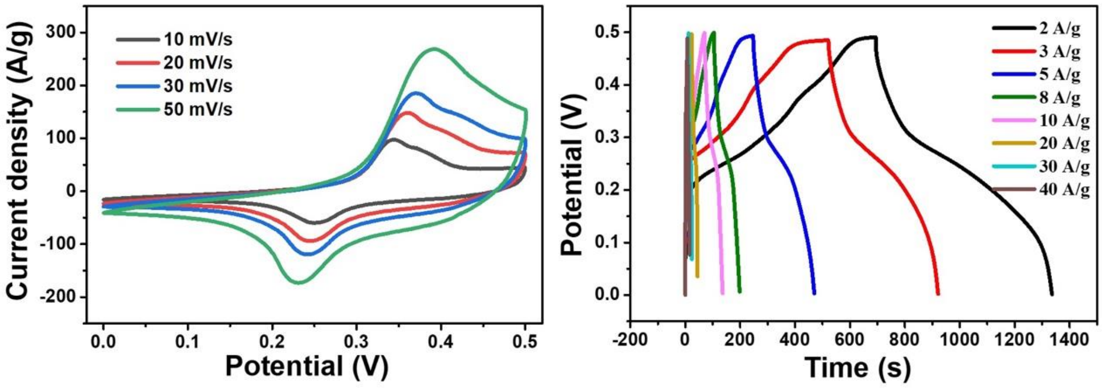

Figure S8. (a) CV and (b) GCD curves of ZCO/BG at different scan rates and current densities, respectively.

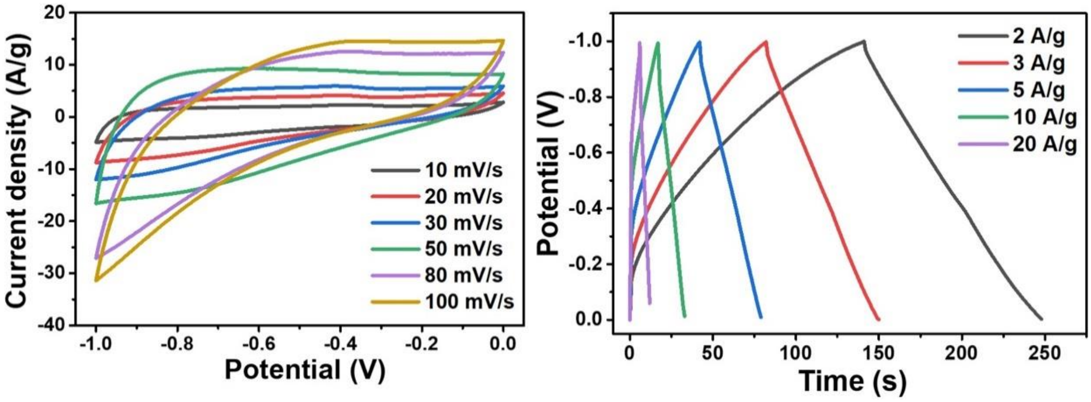

Figure S9. (a) CV and (b) GCD of CS-KOH at different scan rates and current densities, respectively. The specific capacitance at different current densities was of 213.6 F/g at 2 A/g, 201.1 F/g at 3 A/g, 183.3 F/g at 5 A/g, 154.3 F/g at 10 A/g, 117.6 F/g at 20 A/g.

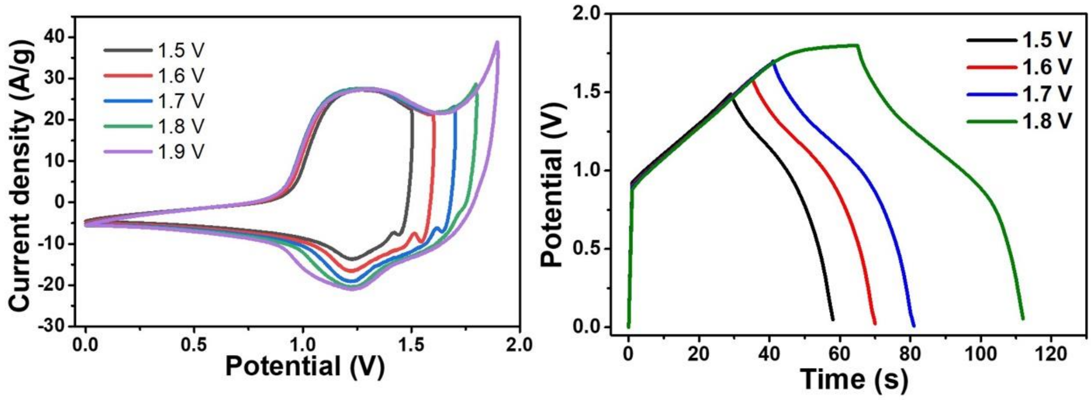

Figure S10. CV and GCD curves at different voltage of ZCO/BG // CS-KOH device at a constant scan rate (100 mV/s) and current density (5 A/g).

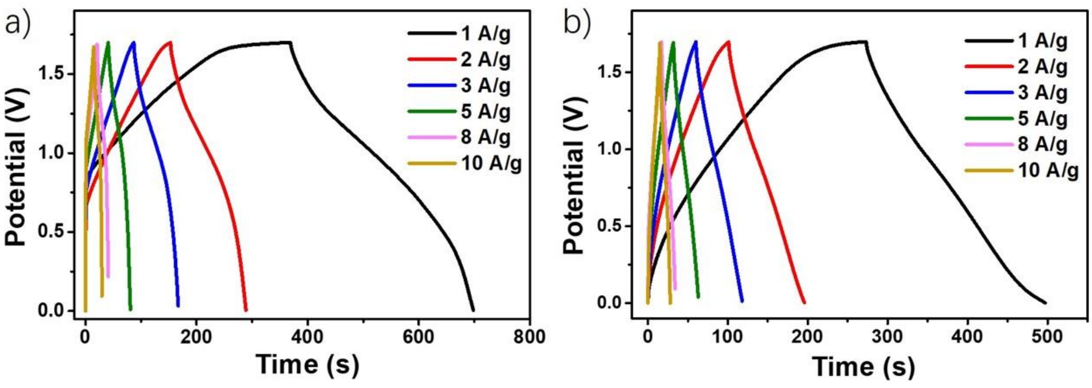

Figure S11. GCD curves of ZCO/BG // CS-KOH (a) and ZCO/NG // CS-KOH (b) at different current densities at 1.7 V.

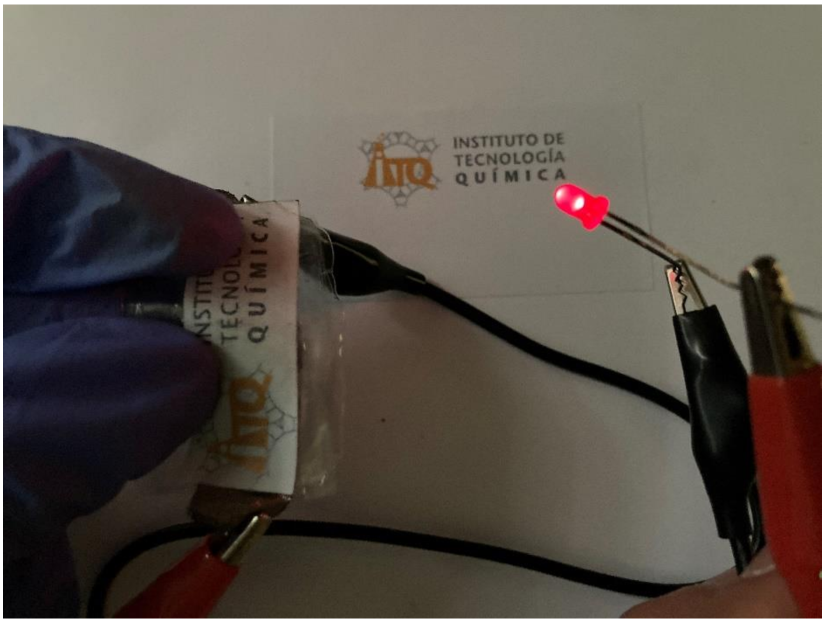

Figure S12. Digital picture of the *quasi*-solid-state, flexible ZCO/BG // PVA-KOH // CS-KOH supercapacitor feeding a red LED operating at a minimum potential of 1.6 V.

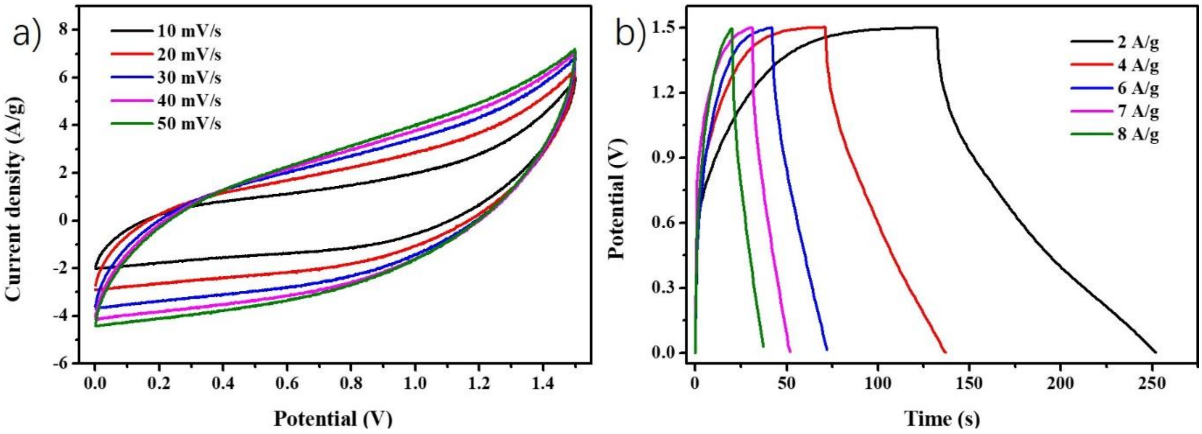

Figure S13. CV (a) and GCD (b) curves of the *quasi*-solid-state, flexible ZCO/BG // PVA-KOH // CS-KOH supercapacitor.

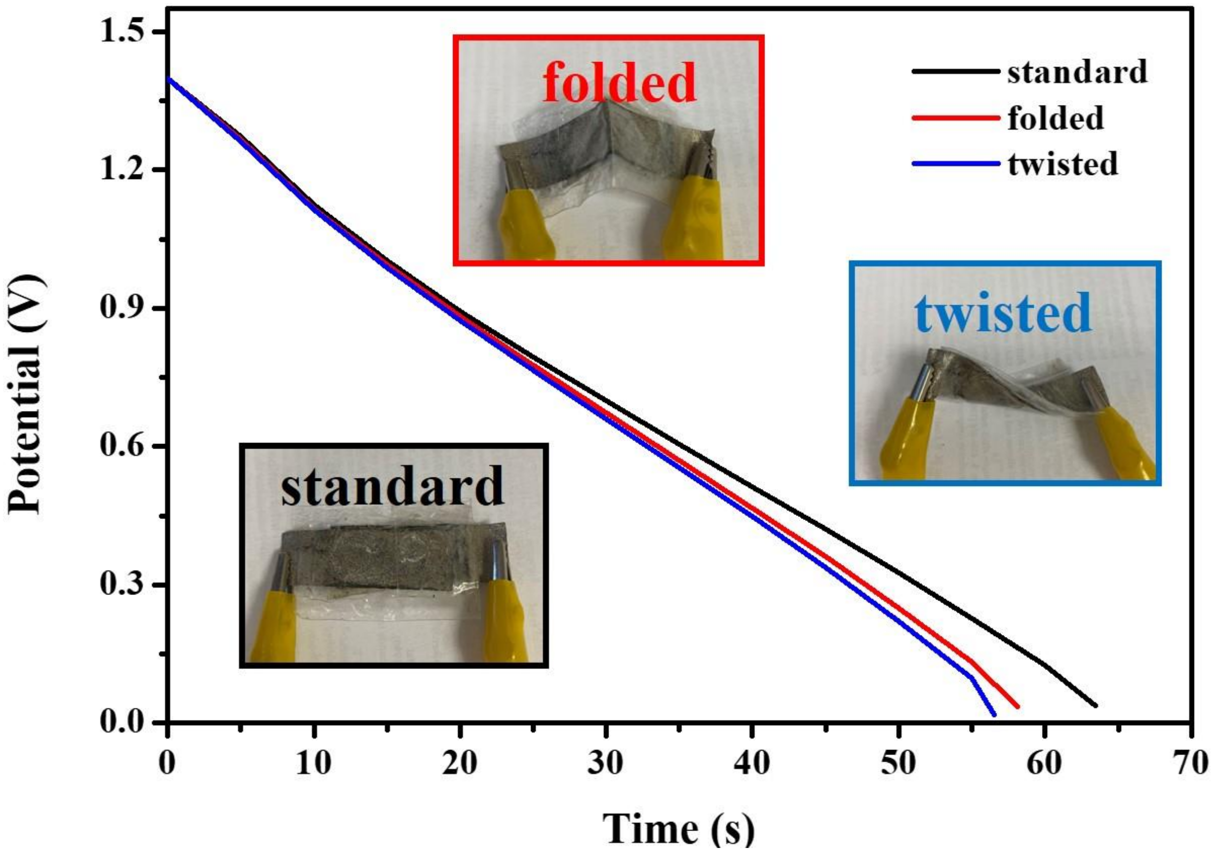

Figure S14. GCD curves at 4 A/g of ZCO/BG // PVA-KOH // CS-KOH under different mechanical stress.

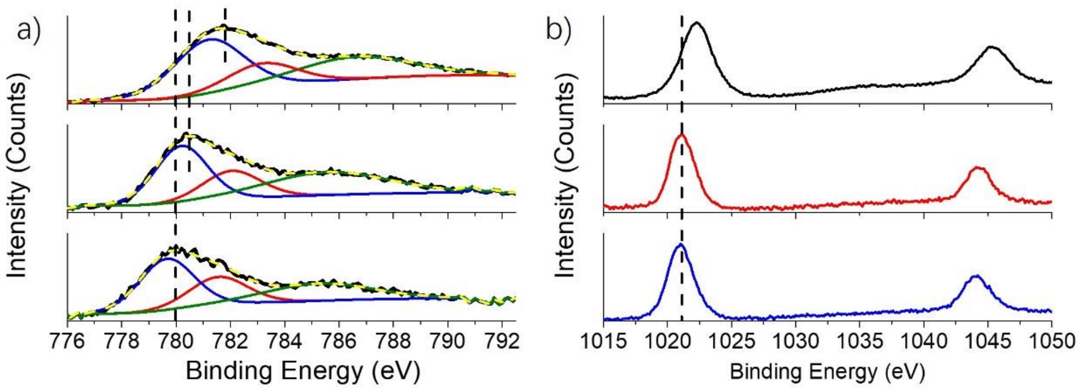

Figure S15. XPS Co 2p (a) and Zn 2p (b) spectra of ZCO/NG and ZCO/BG.

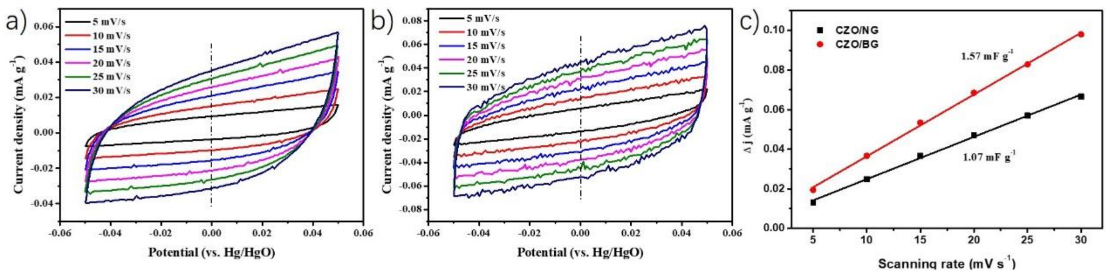

Figure S16. CV at different scan rates of ZCO/NG (a), ZCO/BG (b) and the capacitive current as scan rate function of the samples. The ECSA obtained is indicated.

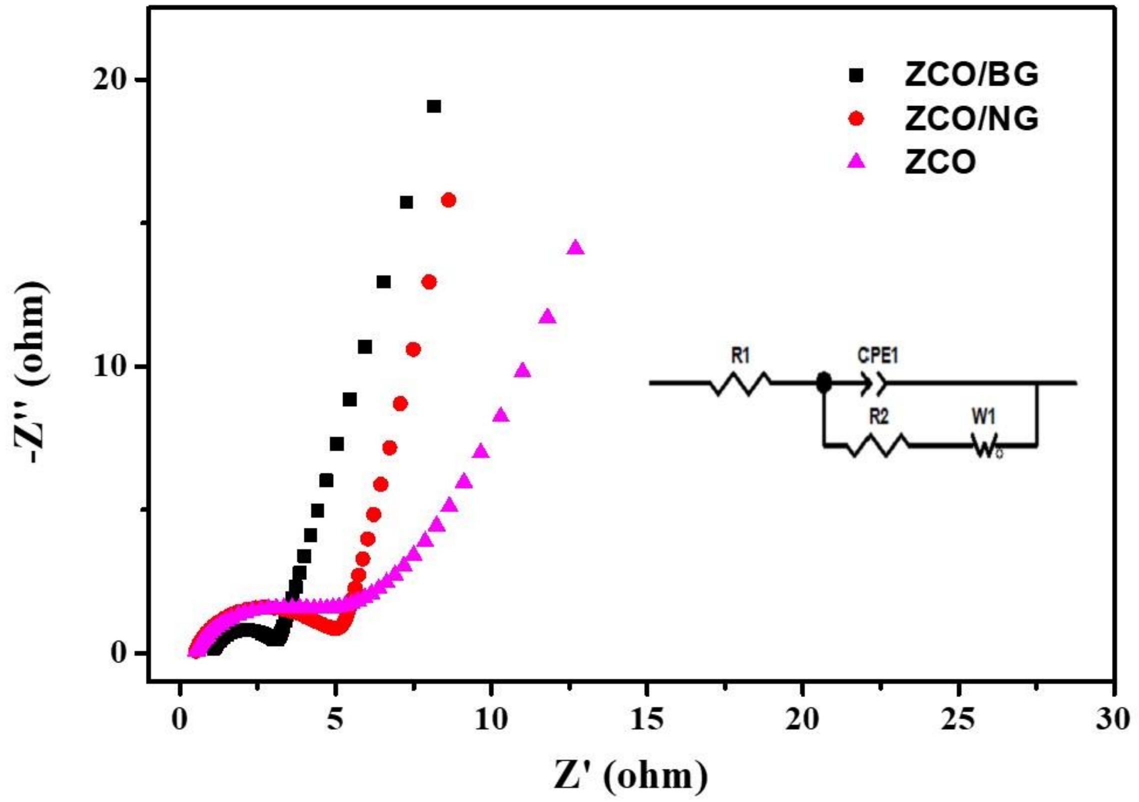

Figure S17. Nyquist plot of ZCO, ZCO/NG and ZCO/BG.

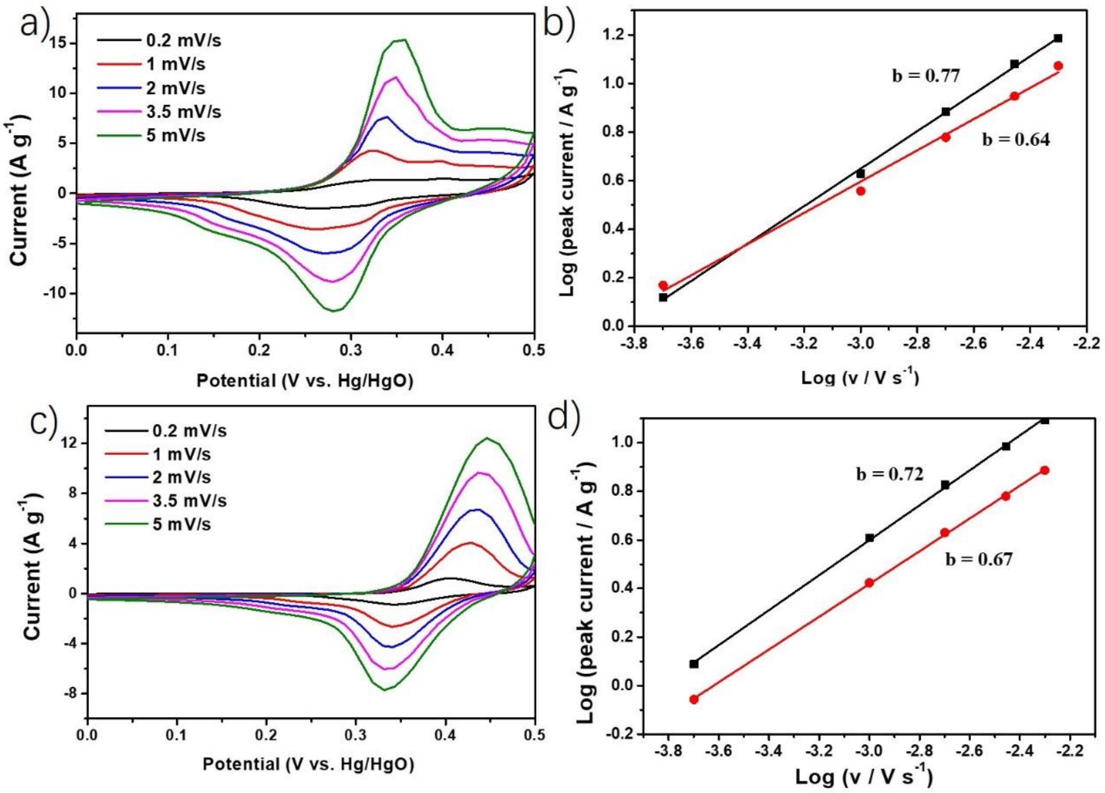

Figure S18. CV curves of ZCO/BG (a) and ZCO/NG (c) at different scan rates. Logaritmic relation between the peak current and scan rate of ZCO/NG (b) and ZCO/NG (d).

References

[1] X. Tian, X. Sun, Z. Jiang, Z.-J. Jiang, X. Hao, D. Shao, T. Maiyalagan, *ACS Appl. Energy Mater.* **2018**, 1, 143.

[2] Z. Gao, L. Zhang, J. Chang, Z. Wang, D. Wu, F. Xu, Y. Guo, K. Jiang, *Appl. Surf. Sci.***2018**, 442, 138.

[3] L. Cheng, M. Xu, Q. Zhang, G. Li, J. Chen, Y. Lou, *J. Alloys and Compd.* **2019**, 781, 245.

[4] G. Wei, Z. Zhou, X. Zhao, W. Zhang, C. An, *ACS Appl. Mat. Interfaces* **2018**, 10, 23721.

[5] L. Niu, Z. Li, W. Hong, J. Sun, Z. Wang, L. Ma, J. Wang, S. Yang, *Electrochim. Acta* **2013**, 108, 666.

[6] H. Yang, X. Zhu, E. Zhu, G. Lou, Y. Wu, Y. Lu, H. Wang, J. Song, Y. Tao, G. Pei, Q. Chu, H. Chen, Z. Ma, P. Song, Z. Shen *Nanomaterials* **2019**, 9, 345.

[7] C. Zhao, L. Lin, S. Tian, P. Nie, X. Xue, H. Wang, T. Xu, L. Chang, *J Inorg. Organomet. Polym. Mater.* **2021**, 31, 3953.
